# Ongoing validation of health-related quality of life instruments

**DOI:** 10.1186/1745-6215-16-S2-O8

**Published:** 2015-11-16

**Authors:** Kim Cocks, Puvan Tharmanathan

**Affiliations:** 1 York Trials Unit, University of York, York, UK

## Background

Validated health-related quality of life (HRQOL) instruments may require updating as they begin to be used in randomised controlled trials (RCTs) containing patient populations and novel drugs that did not form part of the original validation study. Multiple myeloma (MM) is one example where new classes of drugs are rapidly emerging. There are a number of commonly used HRQOL instruments used, some generic and some MM specific but it is unclear how valid existing instruments will be in RCTs of novel agents. We aim to make recommendations for ongoing validation of HRQOL instruments in RCTs applicable to many disease areas using empirical evidence from the case of MM.

## Methods

We searched MEDLINE for RCT's in MM measuring HRQOL conducted in the last 10 years. We also surveyed instrument publications and websites in order to determine whether the instrument was validated for use in the RCT population, how long ago and whether there are provisions for ongoing validation.

## Results

Our search returned 146 abstracts. Validation studies sometimes contained a small sample of sub-populations of a disease (e.g. the EORTC QLQ-MY20 validation sample had 225 newly diagnosed and only 15 relapsed patients).

## Conclusions

Health-related quality of life instruments should be subjected to ongoing validation to ensure they remain relevant in their field and for specific clinical trial patient populations. We discuss simple psychometrics that can be incorporated into the RCT statistical analysis plan and adding debrief questions to the instrument could provide additional insight, particularly in phase II studies of novel drugs.

